# Small fibre integrity and axonal pathology in the rat model of experimental autoimmune neuritis

**DOI:** 10.1093/braincomms/fcae059

**Published:** 2024-03-01

**Authors:** Pia Renk, Melissa Sgodzai, Rafael Klimas, Alina Blusch, Thomas Grüter, Jeremias Motte, Xiomara Pedreiturria, Jeannette Gebel, Philipp Gobrecht, Dietmar Fischer, Ralf Gold, Kalliopi Pitarokoili

**Affiliations:** Department of Neurology, St. Josef-Hospital, Ruhr-University Bochum, 44807 Bochum, Germany; Department of Neurology, St. Josef-Hospital, Ruhr-University Bochum, 44807 Bochum, Germany; Department of Neurology, St. Josef-Hospital, Ruhr-University Bochum, 44807 Bochum, Germany; Department of Neurology, St. Josef-Hospital, Ruhr-University Bochum, 44807 Bochum, Germany; Department of Neurology, St. Josef-Hospital, Ruhr-University Bochum, 44807 Bochum, Germany; Department of Neurology, St. Josef-Hospital, Ruhr-University Bochum, 44807 Bochum, Germany; Department of Neurology, St. Josef-Hospital, Ruhr-University Bochum, 44807 Bochum, Germany; Center for Pharmacology, University Hospital Cologne, 50931 Cologne, Germany; Center for Pharmacology, University Hospital Cologne, 50931 Cologne, Germany; Center for Pharmacology, University Hospital Cologne, 50931 Cologne, Germany; Department of Neurology, St. Josef-Hospital, Ruhr-University Bochum, 44807 Bochum, Germany; Department of Neurology, St. Josef-Hospital, Ruhr-University Bochum, 44807 Bochum, Germany

**Keywords:** small fibres, pain, experimental autoimmune neuritis, autoimmune neuropathies

## Abstract

Experimental autoimmune neuritis is a common animal model for acute human immune–mediated polyneuropathies. Although already established in 1955, a number of pathophysiological mechanisms remain unknown. In this study, we extensively characterize experimental autoimmune neuritis progression in Lewis rats, including new insights into the integrity of small nerve fibres, neuropathic pain and macrophage activation. Acute experimental autoimmune neuritis was induced with P2_53–78_ peptide and consequently investigated using the gait analysis system CatWalk XT, electrophysiological and histopathological analyses, quantitative polymerase chain reaction (PCR), dorsal root ganglia outgrowth studies, as well as the von Frey hair and Hargreaves tests. For the longitudinal setup, rats were sacrificed at Day (d) 10 (onset), d15 (peak), d26 (recovery) and d29 (late recovery). We confirmed the classical T-cell and macrophage-driven inflammation and the primarily demyelinating nature of the experimental autoimmune neuritis. The dual role of macrophages in experimental autoimmune neuritis is implicated by the high number of remaining macrophages throughout disease progression. Furthermore, different subpopulations of macrophages based on *Cx3-motif chemokine receptor 1* (*Cx3cr1)*, *platelet factor 4 (Pf4)* and *macrophage galactose-type lectin-1 (Mgl1)* expressions were identified. In addition, modulation of the sensory system in experimental autoimmune neuritis was detected. An outgrowth of small fibres in the plantar skin at the onset and peak of the experimental autoimmune neuritis was evident parallel to the development of acute hyperalgesia mediated through transient receptor potential vanilloid 1 modulation. Our data depict experimental autoimmune neuritis as a primary demyelinating disease with implicated axonal damage, a small unmyelinated fibre impairment throughout the disease progression course, and underline the pivotal role of macrophages in the effector and during the recovery stage.

## Teaser

Experimental autoimmune neuritis is a widely used animal model for immune-mediated neuropathies. Renk *et al*. reported a thorough longitudinal characterization of the disease, revealing novel findings including the altered integrity of small fibres correlating with altered pain sensation and the identification of different macrophage subtypes during the recovery phase correlating with improved regenerative capacity.

## Graphical Abstract

**Graphical Abstract:**
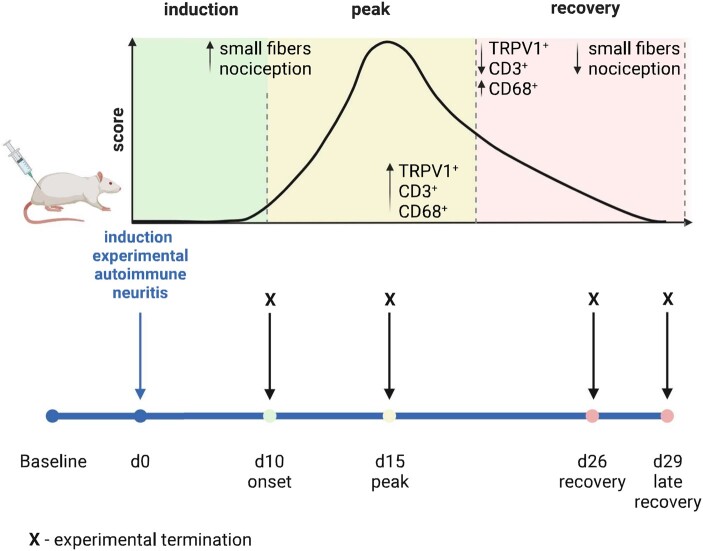

## Introduction

Autoimmune-mediated polyneuropathies are a heterogenous group characterized by an autoimmune reaction directed against nerve tissue in the peripheral nerve system. Guillain–Barré syndrome (GBS) represents the most acute form of autoimmune-mediated polyneuropathies with an incidence rate of 0.8–1.9 cases per 100 000 people per year in North America and Europe.^1,2^ The prevalence of the chronic form, chronic inflammatory demyelinating polyradiculoneuropathy (CIDP), ranges from 0.8 to 8.9 cases per 100 000 people.^3^ Clinical manifestations of GBS and CIDP are proximal and distal weakness, sensory deficits and areflexia. The pathogenesis is characterized by an inflammatory reaction directed primarily against myelin and axonal structures mediated by a cellular and humoral immune response. Both motor and sensory axons are typically affected by demyelination and axonal damage. First-line therapy for CIDP consists of intravenous immunoglobulins, corticosteroids and plasma exchange with a response rate of up to 75%.^4-9^ The high percentage of non-responders and 50% of patients experiencing clinical relapses after treatment, as well as early axonal degeneration for the typical CIDP with a progressive disease course, highlights the necessity of new therapies and strategies.^2,10-12^

To research therapeutic options and gain a deeper understanding of the underlying immune mechanism of inflammation and degeneration of GBS/CIDP, the experimental autoimmune neuritis (EAN) model was established. The EAN resembles the clinical course, electrophysiological and histological characteristics of acute inflammatory demyelinating polyradiculoneuropathy, and is widely accepted as the corresponding animal model. Active induction is achieved by immunization with peripheral myelin proteins such as P0, P2 or PMP22 emulsified in complete Freund’s adjuvant, resulting in an acute monophasic EAN. This is driven by the infiltration of T cells and macrophages into the peripheral nerve tissue.^13^ Time-dependent histopathological changes in EAN rats were identified by Tomikawa *et al*.^14^ However, no longitudinal study covers more aspects of EAN, such as small fibre involvement, pain sensation and an *in vivo* correlation with T-cell and macrophage activations.

Macrophages can be divided into pro-inflammatory M1 macrophages and anti-inflammatory M2 macrophages. Studies reported the importance of macrophages on the development and progression of EAN based on their inflammatory properties.^15-17^ T-cell–induced macrophage-mediated demyelination in EAN is the primary cause of clinical manifestations (flaccid tail, muscle weakness and mild-to-moderate paraparesis). In contrast, axonal damage is considered a secondary effect. Previous research showed a high variability of reported severity of axonal degeneration.^18^ Information regarding the integrity of small nerve fibres in the context of EAN is widely missing. Alterations are reported and can be associated with sensory dysfunction and pain.^19,20^ A development of EAN-induced hyperalgesia was previously identified from our group in EAN, indicating the involvement of the small nerve fibres.^21^

This study aimed to provide for the first time an in-depth analysis of EAN progression in Lewis rats covering histopathological and electrophysiological alterations, behavioural pain and possible molecular pathway analysis, as well as providing insight into new aspects such as the specification of infiltrating macrophages, small nerve fibre integrity and clinical manifestations assessed by the CatWalk XT.

## Materials and methods

### Animals and experimental design

All experiments were approved by the European Communities Council Directive on 22 September 2010 (2010/63/EEC) for the care of laboratory animals and with local government authorization (Landesamt für Natur, Umwelt und Verbraucherschutz North Rhine-Westphalia; Az.: 81-02.04.2018.A177). Female Lewis rats, ranging from 6 to 8 weeks, were purchased from Charles River (Sulzfeld, Germany) and housed under standardized, pathogen-free conditions in our local animal facility (Medical Faculty, Ruhr-University Bochum) in cages provided with food and water available ad libitum. Eight rats resided in the animal facility of the chair of cell physiology (Biological Faculty, Ruhr-University Bochum) under the same conditions (TVA Nr 81-02.04.2018.A177). Upon arrival, animals could acclimatize for at least 1 week, and their weight ranged between 140 and 160 g.

### Induction of EAN and assessment of the clinical score

Before EAN induction, animals were randomly distributed into control and EAN groups. EAN was induced using an immunization paste consisting of the neuritogenic P2 peptide, corresponding to the amino acids 53–78 of the rat myelin P2 protein, synthesized by Genosphere Technologies (France, Germany). A total of 300 µg P2 peptide was emulsified in complete Freund’s adjuvants containing 1 mg/ml *Mycobacterium tuberculosis* H37RA (Difco). After xylazine and ketamine anaesthetization (CP-Pharma), the suspension was subcutaneously injected into the tail base. Post-immunization, the animals were daily weighed and scored using the following EAN score system: 0 = normal; 1 = reduced tail reflexes; 2 = tail paralysis/impaired righting; 3 = absent righting; 4 = ataxic gait/abnormal paw position; 5 = mild paraparesis; 6 = moderate paraparesis; 7 = severe paraplegia; 8 = tetraparesis; 9 = moribund and10 = death. The experimenters evaluating the clinical course and performing all the following experiments were blinded for the form of immunization and the group of animals analysed.

### von Frey hair test

Assessment of the 50% paw withdrawal threshold was estimated with the von Frey (vF) hair test to evaluate mechanical allodynia/nociception. Tests with monofilaments (BioSebLab, France) were performed on rats previously placed in a plastic box with a customized plexiglass platform of 3-mm thickness and 1.5-mm diameter holes in a grid pattern. To prevent disturbances in the behaviour, animals could habituate for 30 min in the test chambers, and the surface of the platform was obscured to disguise the experimenter’s movement. Monofilaments with a size of 3.84–5.46 corresponding to a force of 0.6–26 g were applied consecutively on the mid-plantar skin of the right hind (RH) paw. Each paw was tested at least six times with an application time of 8 s. The 50% paw withdrawal threshold was calculated according to a modified up-and-down method. A blinded experimenter performed tests to assess allodynia/hyperalgesia over EAN progression on Days 0, 8, 13, 22 and 26 post-immunization (p.i.).

### Hargreaves test

The thermal allodynia/nociception was detected by recording the paw withdrawal time, assessed via the Hargreaves test (Ugo Basile). Animals were placed into plastic chambers on a framed glass panel and allowed to acclimate for 10–15 min. The emitter vessel was positioned directly under the centre of the RH paw, and the withdrawal time in response to the emitted infrared light was automatically recorded. The maximum emitting time was set to 20 s to prevent potential burns. Each animal was tested at least five times with a few seconds’ rest in between. Tests were carried out on Days 0, 7, 12, 14, 16, 19, 23, 27 and 29  days post-immunization (dpi) at the chair of Cell Physiology (Biological Faculty, Ruhr-University Bochum).

### Nerve conduction studies

Nerve conduction studies (NCSs) were performed on Days 8, 15 and 26 p.i. to detect alterations in the motor fibre conduction of the sciatic nerves by determining the motor nerve conduction velocity (MNCV) and F-wave latency. For the duration of the examination, animals were anaesthetized as described above. NCV and F waves were recorded by using a fully digital Keypoint apparatus (Dante, Skovlunde, Denmark). Then, paired needle electrodes were inserted into the right sciatic notch for proximal stimulation and the right malleolus for distal stimulation. Stimulation of 0.05-ms supramaximal rectangular pulses induced compound muscle action potentials (CMAPs), measured with a recording needle electrode placed s.c. over the dorsal foot muscles. A grounding electrode was incorporated into the setup between the stimulating and the active recording electrodes. The NCV was calculated by dividing the distance between both electrodes by the latency difference. A minimum of 15 F waves, evoked by stimulating the electrode in the popliteal fossa, were recorded for the RH paw.

### CatWalk XT

Locomotor activity was assessed using the automated gait system CatWalk XT (Noldus, Wageningen, Netherlands). Therefore, the animals walked along an enclosed corridor with a glass plate as red and green LED lights illuminated the floor. A high-speed colour camera recorded the contact area of the paws and the contour of the animals, allowing multiple parameters to be evaluated. The following parameters were analysed by using the CatWalk XT 10.6 software: RH paw–left front (LF) paw coupling, RH stride length, step regularity index (SRI), print area and print width. The animals were trained to achieve consecutive uninterrupted runs before the data acquisition process. Each animal was recorded for three continuous runs on Days 0, 7, 12, 14, 16, 19, 23, 27 and 29 p.i.

### Toe spreading

After EAN induction, motor function was additionally assessed by determining the ability of the rats to spread their toes. Therefore, rats were lifted from the ground to photograph the left hind paw on various days before and after EAN induction (0, 7, 12, 14, 16, 19, 23, 27 and 29). The toe spreading distance between the first and the fifth toe was then measured. The experimenter performing the toe spreading experiment was blinded for the treatment during this experiment. Data represent mean ± SEM per experimental group. Statistical significances of inter-group differences were evaluated using two-way ANOVA, followed by the Holm–Sidak *post hoc* test.

### Histopathological analysis

The experimenter was blinded for the imaging and analysis of the immunohistochemistry.

### Dorsal root ganglia

Cervical, thoracic and lumbar dorsal root ganglias (DRGs) were dissected and washed with phosphate buffered saline (PBS). The DRGs were transferred in 4% paraformaldehyde (PFA) for at least 16 h and subsequently cryoprotected in 30% sucrose at 4°C for up to 5 days. Cryosections of DRGs (8 µm) were sliced using the cryostat Microm HM550 (Thermo Fisher Scientific, USA) and stained with a primary antibody against transient receptor potential cation channel subfamily V member 1 (TRPV1; 1:500, Thermo Fisher Scientific, RRID: AB_1961203). The secondary antibody anti-rabbit Alexa 568 (1:1000, Invitrogen, A-11036) was used to visualize TRPV1 staining. Cell nuclei were stained with 4′,6-diamidino-2-phenylindole (DAPI) Fluoromount-G (Biozol). Fluorescence signals were detected and recorded using an inverted fluorescence microscope (Elyra PS.1 with LSM 880, Zeiss). Two to three DRGs of each section (cervical, thoracic and lumbar) of each rat were imaged (minimal technical *n* = 6, maximal technical *n* = 9) and analysed using the image analysis softwares ImageJ (National Institutes of Health, Bethesda, MD, USA) and ZEN Blue 3.1 lite (Zeiss, Oberkochen, Germany).

### Sciatic nerve

Dissected left-side sciatic nerves were divided into three parts of equal length, embedded and cut into cryosections (8 µm). Cross-sections were stained with antibodies against SCG10 (1:1000, Novus Biologicals, RRID: AB_10011569) and GAP43 (1:1000, Invitrogen, custom-made), expressed in injured axons, to assess axonal injury and regeneration. To determine T-cell and macrophage infiltration, primary antibodies CD3 (1:100, Invitrogen, RRID: AB_468845) and CD68 (1:100, Hycult Biotech, RRID: AB_10130957) were used. A primary antibody against TRPV1 (1:500, Thermo Fisher Scientific RRID: AB_1961203) was used to analyse nociception. Secondary antibodies against mice or rabbits coupled to Alexa Fluor 555 (1:1000, BioLegend, RRID: AB_2563179) were applied to visualize the corresponding structures. FluoroMyelin™-Red Fluorescent Myelin Stain (1:300, Thermo Fisher Scientific, RRID: AB_2572213) was used to assess the demyelination of the nerve tissue. Three nerve sections of each animal were recorded (technical *n* = 9) and analysed using the function colour threshold and plugin cell counter in ImageJ (National Institutes of Health).

### Skin

Skin biopsies were taken from the front and hind paw footpads and washed with PBS, fixed in 4% PFA for 30 min and subsequently cryoprotected in 30% sucrose. Cryosections (20 µm) were stained against TRPV1 (1:500, Thermo Fisher Scientific, RRID: AB_1961203), PGP9.5 (1:800, Abcam, RRID: AB_1269733) and beta-III-tubulin (1:2000, BioLegend, RRID: AB_12313773) and visualized with Alexa Fluor 555 (1:1000, BioLegend, RRID: AB_2563179), Alexa Fluor 488 (1:1000, BioLegend, RRID: AB_2563044) or Alexa Fluor 594 (1:1000, Thermo Fisher) to evaluate axonal degeneration and alteration of nociception. Axon density in the stratum spinosum of the most distal footpad innervated by the sciatic nerve was determined according to a method established in different publications from different labs.^22-25^ Therefore, footpads were cut into 20-µm sections, yielding ∼100 sections/footpad. Every fourth section (10 in total) was then immunohistochemically stained against βIII-tubulin to visualize axon distribution within the stratum spinosum of the entire footpad. All axons innervating the stratum spinosum from the stratum basale of each section were counted. The stratum basale was visualized with DAPI counterstaining. The length of the axons within the stratum spinosum was not taken into consideration. Per the evaluated animal, the average axon number of the 10 evaluated sections was determined. These numbers are represented as a dot [control (CTR)], as a square [Peak (P) EAN], as a triangle [Recovery (R) EAN] or as a diamond [late recovery (LR) EAN] in the respective graph.

In addition, cell nuclei were stained with DAPI Fluoromount-G (Biozol). Beta-III-tubulin and TRPV1-positive structures in the stratum spinosum layer were analysed in terms of the quantity or positive area.

### *In vitro* DRG outgrowth study

Sensory neuron axon growth of DRGs was evaluated by assessing the length of outgrown axons. Lumbar DRGs were dissected and cultivated for 3 days in a neurobasal medium infused with B27 (2%, Gibco), fetal horse serum (2%, PAN-Biotech), glutamine (1%, Thermo Scientific), gentamycin (0.5%, Gibco) and neural growth factor (10 ng/ml, Sigma-Aldrich) on poly-d-lysine (0.2 mg/ml, Sigma-Aldrich) and laminin (1 µg/ml, Sigma-Aldrich)–coated slides in a 5% CO_2_-humified atmosphere. Axonal growth was determined after 72 h by fixation in 4% PFA and stained with primary antibody beta-III-tubulin (1:2000, Sigma-Aldrich, RRID: AB_262133). Imaging was performed with an inverted fluorescence microscope (BX51, Olympus). Axon lengths were measured with the NeuronJ plugin for ImageJ. Three to four DRGs of each rat were recorded and analysed.

### Quantitative PCR

Right-side sciatic nerves were dissected, immediately flash-frozen in liquid nitrogen and stored at −80°C. The frozen nerve tissue was transferred into a reaction vessel containing TRIzol™ (Thermo Fischer Scientific) and stainless-steel beads of 5-mm diameter (Qiagen) and lysed and homogenized via the Tissue Lyser II (Qiagen). RNA isolation was achieved by using the RNeasy Plus Mini kit (Qiagen) and transcribed into cDNA using the Go Script Reverse Transcription Mix Oligo(dT) and random primers (Promega) according to the manufacturer’s protocol. Analysis of target genes’ mRNA expression was realized using the GoTaq® qPCR Master Mix (Promega) and target-specific primers (Microsynth). Targeted genes were chosen according to their role in neuroinflammation, neurodegeneration, nociception and the identification of macrophage populations. *Ifng*, *Il10*, *Trpv1*, *Calca*, *Gap43*, *Mgl1* (also known as *Clec10a*), *Cx3cr1* and *Pf4* were covered (Supplementary Table 1). Emission was detected by the QuantStudio 3 Real-Time-PCR system (Thermo Fischer Scientific). qPCR runs were composed of different stages: 95° for denaturation, 60°C for annealing/extension and repeated for 40 cycles. A melting curve analysis was performed to authenticate the amplification of the specific target genes and check for primer-dimer artefacts. The Pfaffl analysis was used for calculating the relative target expression taking the primer’s efficacy into account.^26^  *Gapdh* and *Actb* were reference genes to normalize the target gene expression levels. Relative target gene expression is described as fold-change relative to the corresponding experimental control group. All experiments were carried out in duplicates, and the mean Ct value was used in the equation.

### Statistical analysis

Statistical analyses were performed using GraphPad Prism 9 (GraphPad Software Inc.) or Sigma STAT3.1. The normality of data was analysed before testing. Based on the normality, parametric or non-parametric tests were carried out. Histological and quantitative reverse transcription - PCR (qRT-PCR) experimental data were analysed via one-way ANOVA for multiple comparisons or unpaired two-tailed *t*-tests. Electrophysiological and behavioural test results were compared using ANOVA in combination with Tukey’s multiple comparison test and Holm–Sidak’s multiple comparison test, respectively. Probability level (*P*-value) *P* ≤ 0.05 was considered significant and is indicated as *P ≤ 0.05, **P ≤ 0.005, ***P ≤ 0.001 and ****P ≤ 0.0001. Data are presented as means ± standard deviation.

## Results

### Acute motor manifestations

#### Clinical scoring

**Figure 1:**
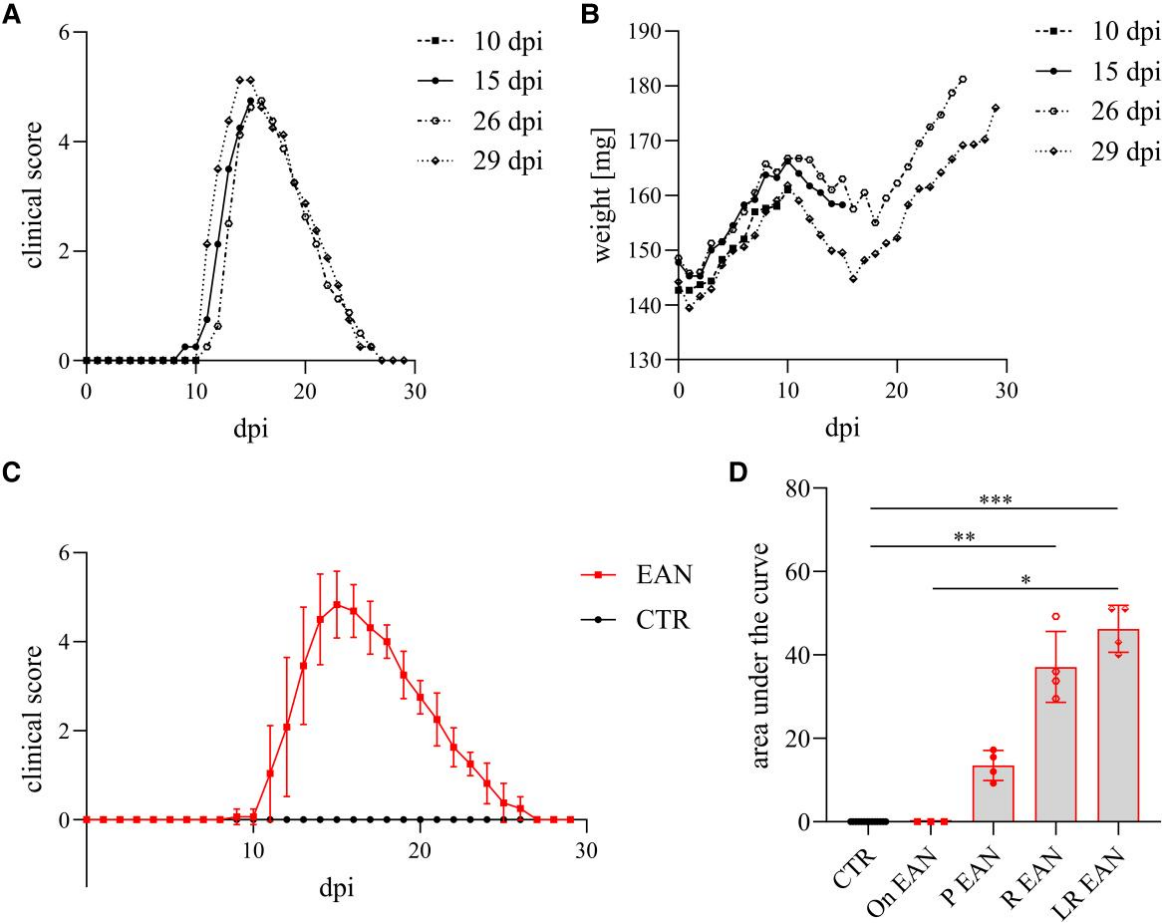
**(A) The progression of the clinical course of EAN rats.** (**B**) The weight of rats reduces concordantly with the worsening of clinical manifestations. Each point presents a mean value. Group 10 dpi includes a *n* = 3; Group 15, 26, and 29 dpi a *n* = 4. (**C**) An overview of the clinical course of the EAN and CTR groups. (**D**) An AUC analysis of EAN progression shows a significant increase in EAN rats over the disease course (AUC and Kruskal–Wallis test followed by Tukey’s multiple comparison test, *P*-value < 0.0001–0.05). Days 0–10 p.i. include a *n* = 15, Days 11–15 p.i. *n* = 12, Days 16–26 p.i. *n* = 8 and Days 27–29 p.i. *n* = 4. AUC, area under the curve; On, onset.

According to the EAN score (see the Materials and methods section), all P2-immunized animals developed clinical motor neuritis around Days 10–12 p.i. (Fig. 1A and C). Motor symptom severity peaked on Day 15 p.i. (mean values of score: *P*-value < 0.0001), lasting for a maximum of 2 days and a subsequent transition into remission. Full recovery was achieved in all animals on Day 27 p.i. All EAN animals suffered from weight loss during the phase with the maximum severity, whereas weight gain was documented as coinciding with the disease progression in the recovery phase (Fig. 1B). Rats immunized with complete adjuvant in PBS without the P2 peptide (control group) showed no signs of motor dysfunction (Fig. 1C and D) and weight loss.

#### CatWalk

**Figure 2:**
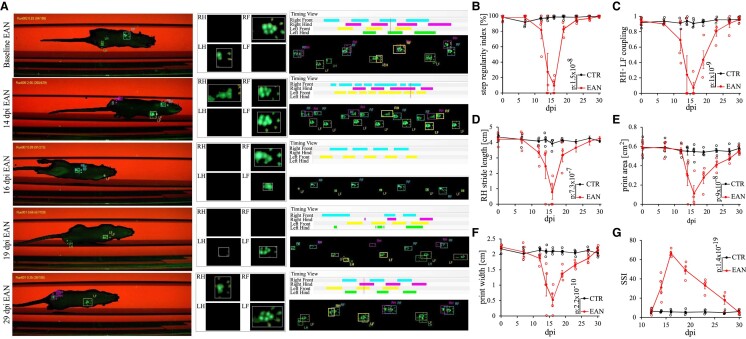
**Representative pictures of the CatWalk test (A) and corresponding parameters (B–F).** The CatWalk analysis identified on Day 16 p.i. (two-way ANOVA followed by Tukey’s multiple comparison test: *P*-value < 0.0001) as the peak of clinical manifestations with a subsequent recovery fully achieved on Day 29 p.i. Three consecutive runs for each animal (*n* = 4/group) for each time point were recorded. (**G**) The static sciatic index measured via toe spreading showed a prolonged recovery (two-way ANOVA followed by Tukey’s multiple comparison test: *P*-value < 0.0001). SSI, static sciatic index.

To obtain a precise objective evaluation of further clinical EAN manifestations, including gait stability, the CatWalk test was performed in EAN rats (Fig. 2). Initial characteristic clinical manifestations of neuritis in P2-immunized rats were recorded simultaneously with the EAN score on Day 12 p.i. EAN rats showed a decreased SRI, reflected by the loss of conjoint steps of the front and hind paws and diminished stride length, reaching its maximum on Day 16 p.i., 1 day after the maximum of the motor EAN score before progressing into the recovery phase. On Day 29 p.i. 2 days later, compared with the EAN score, no alterations in the parameters mentioned above were recorded, depicting the reconstitution of the original condition. The print area and print width parameters showed a similar progression with slightly prolonged remission. We saw a faster improvement of parameters regarding the motor nerves solely (SRI, RH–LF coupling and RH stride length), which implies that sensory dysfunction in the context of EAN, such as sensory-mediated ataxia, altered the parameters influencing the appearance of the paws. The acute worsening and subsequent amelioration of analysed parameters mirrored the disease progression assessed via clinical scoring, affirming the accuracy of the CatWalk for further EAN investigations with the potential of distinguishing motor nerve–mediated from sensory nerve–mediated clinical symptoms.

#### Toe spreading

Further assessment of motor function was acquired by analysing the toe spreading reflex of the rats’ left hind paws (Fig. 2G). Reduced toe spreading ability in EAN-affected animals was first detected at Day 12 p.i., reaching its maximum at Day 16 p.i. followed by a gradual remission to a full recovery at Day 29 p.i. The progression mirrors the parameters of print width and print area, assessed in the CatWalk test, supporting our assumption that sensory ataxia affects the paw appearance in an EAN setting.

### Large fibre demyelination and degeneration

#### Electrophysiological measurements

Demyelination and axonal degeneration were assessed by NCSs of the sciatic nerves at different time points over the disease course (Supplementary Fig. 1). The significant decrease in the MNCV compared with the control group indicates the impaired myelin integrity of the distal segment of the motor nerve fibres. Reduced MNCVs were recorded on Days 8 (mean values: P & R: *P*-value = 0.0127), 15 (P & R: *P*-value < 0.0001; LR: *P*-value = 0.0075) and 29 (LR: *P*-value = 0.0065) p.i. The lowest MNCV recorded on Day 15 p.i. coincided with the peak of motor symptoms. Decreased MNCVs, recorded at the latest examined time point, showed improvement but did not reach their original state implicating a degree of remaining damage at this time point, even though complete functional recovery had occurred. Significantly prolonged F waves were detected in EAN animals on Day 15 p.i. (mean values: P & R: *P*-value = 0.0056), indicating additional demyelination of the proximal nerve segment. The reduction of CMAPs of the distal (P & R: *P*-value = 0.0013) and proximal (P & R: *P*-value = 0.0042) parts of the nerve points to a proximal conduction block and possible axonal damage.

#### Histological signs of demyelination

FluoroMyelin™ was used to visualize the myelination status of the sciatic nerve. A decrease of the myelinated area was detected at all examined time points (15 dpi: *P*-value = 0.0571; 26 dpi: *P*-value = 0.0571; 29 dpi: *P*-value = 0.0286) except for Day 8 p.i. (data not shown) in EAN-affected animals (Supplementary Fig. 2). Thus, demyelination persisted on an immunohistochemical level. The progression pattern bears a close resemblance to the MNCV and CMAP decrease by reaching its height at peak (15 dpi) with subsequent amelioration but no reversion to the initial state.

#### Implication of axonal regeneration

**Figure 3:**
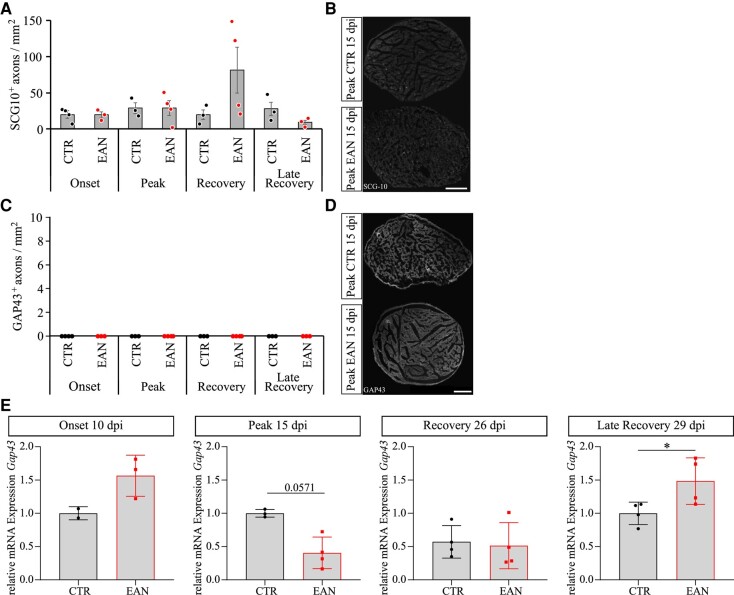
**An immunohistochemical analysis of SCG-10, GAP43 and qPCR results of GAP43 in sciatic nerves of Lewis rats.** (**A**) SCG-10 shows no significant regulation over EAN progression except for two animals on 26 dpi. GAP43 has a dynamic expression on mRNA level (unpaired *t*-test or Mann–Whitney test according to probability distribution: *P*-value_peak_ = 0.0571; *P*-value_late recovery_ = 0.00286) (**E**), whilst no differences were visible on histological level (**C**). Representative pictures of SCG-10 (**B**) and GAP43 (**D**). Each data point represents the value for one animal. The scale indicates 250 µm.

SCG-10 and GAP43, markers expressed in regenerating nerves after axonal damage, were investigated to examine further possible axonal damage in sciatic nerve tissue (Fig. 3). Two of four animals on Day 26 p.i. displayed an increased density of SCG10-positive axons, indicating a prior occurring axonal degeneration. *Gap43* showed a dynamic regulation over EAN progression on the mRNA level of sciatic nerve tissue. Significant upregulation was detected on Day 29 p.i. (*P*-value = 0.0286), whereas *Gap43* showed a trend of downregulation at peak (*P*-value = 0.0571), a further indicator for axonal damage. Contrary to these results, no changes in GAP43 on protein level in sciatic nerves at any examined time point were detected.

#### Enhanced DRG outgrowth *in vitro*

**Figure 4:**
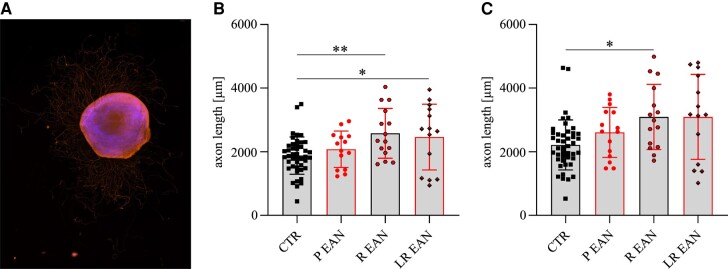
**Results of the *in vitro* DRG outgrowth assay.** (**A**) A representative picture of a DRG. The scale indicates 400 µm. (**B**) The mean value of 10 longest outgrown axons of each DRG identified a higher regeneration potential in the recovery (Kruskal–Wallis test: *P*-value = 0.007) and the late recovery phases (*P*-value = 0.0422). (**C**) The mean value of the singular longest axon of each DRG showed a significant increase in length in the recovery phase (Kruskal–Wallis test: *P*-value = 0.0244). A total of four DRGs per rat were recorded with a *n* = 4/group. Non-adherent DRGs were excluded. The scale indicates 400 µm.

Somata of sensory afferent fibres are located in the DRGs. An *in vitro* neurite outgrowth study was performed to evaluate these fibres’ regeneration potential. DRGs of EAN rats showed a significant increase in axon length in the recovery phase on Days 26 and 29 p.i., pointing to an enhanced regeneration potential in the later stage of EAN progression (Fig. 4).

### Neuroinflammation and immune cell infiltration

#### Upregulation of inflammatory cytokines

The relative mRNA expression of *Ifng* and *Il10*, effector cytokines of Th1 Th2 immune responses, respectively, was investigated throughout the EAN (Supplementary Fig. 3A and B). The pro-inflammatory marker *Ifng* was upregulated at the peak and recovery phases, with ∼45-folded expression on Day 15 p.i. (*P*-value = 0.0571) compared with the control group. Even though still significantly upregulated, the *Ifng* levels on Days 26 (*P*-value = 0.0286) and 29 p.i. (*P*-value = 0.0286) were diminished in comparison. No alteration in mRNA expression was detected on Day 10 p.i. Relative mRNA expression of *Il0* fluctuates over disease progression. The anti-inflammatory marker *Il10* is upregulated on Day 29 p.i. (*P*-value = 0.0286) pointing to molecular mechanisms occurring to prevent the (new) outbreak of neuritis, whereas the slight trend of the increased expression fold on Day 15 p.i. is most likely due to the progression into the recovery and thus Th2 driven. A subsequent trend of downregulation of *Il10* on Day 26 p.i. was detected.

#### T cells

T-cell infiltration is a characteristic feature of EAN. Therefore, the Pan T-cell marker CD3 was histologically examined. CD3-specific cells were detected in cluster-like arrangements in significantly higher numbers in the sciatic nerve of EAN animals. The infiltration rate reached its peak on Day 15 p.i. (*P*-value = 0.0008) before its quick reduction assessed at later examined time points (Supplementary Figs 3C and 4A), mirroring the time-dependent upregulation of *Ifng*, indicating the Th1-specific upregulation of pro-inflammatory cells and cytokines.

#### Macrophages

**Figure 5:**
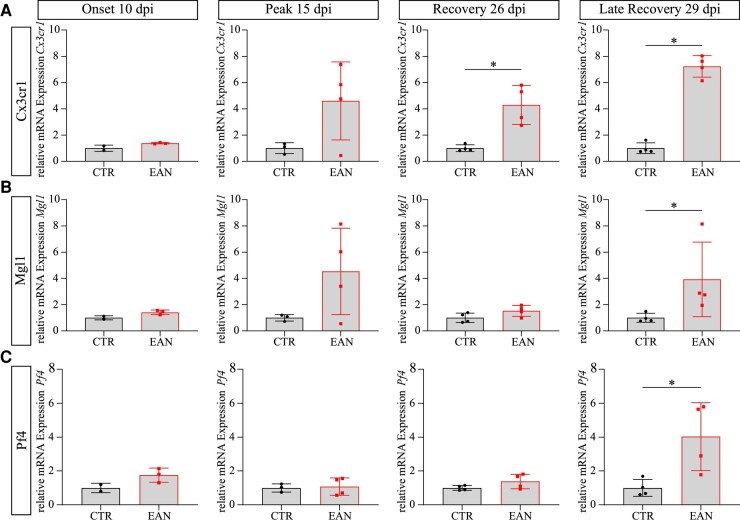
**Subpopulations of infiltrated macrophages in the sciatic nerve.** According to the homogeneity of variances, Welch’s *t*-test or Mann–Whitney test was performed. (**A**) Relative *Cx3cr1* expression is upregulated at recovery (*P*-value = 0.0286) and late recovery (*P*-value = 0.0286). (**B**) *Mgl1* expression is significantly upregulated at late recovery (*P*-value = 0.0286). (**C**) *Pf4* expression is significantly increased at late recovery (*P*-value = 0.0286), indicating the involvement of different macrophage subtypes in the EAN (*n* = 4 in Peak EAN, Recovery CTR & EAN, Late Recovery CTR & EAN; *n* = 3 in Onset EAN, Peak CTR; *n* = 2 in Onset CTR). Quantitative PCR was performed with duplicates per animal. Each data point represents the value for one animal.

The general macrophage marker CD68 was used to identify infiltrating macrophages. Macrophages are significantly enriched in the nerves of P2-immunized rats at all examined time points (Supplementary Figs 3D and 4D). The highest number of CD68-positive cells was found at the peak time point (15 dpi, *P*-value = 0.0034). The number of infiltrating macrophages decreased with the progression of EAN but remained at a higher amount in comparison with T cells. The persisting increased macrophage number in later phases indicates different subpopulations with different immunological properties and the role of macrophages in EAN recovery. To distinguish between the overall macrophage population, different markers were analysed at the mRNA level to identify macrophage subtypes (Fig. 5). *Cx3cr1*, *Pf4* and *Mgl1* were evaluated for the first time in EAN. The high expression of *Cx3cr1* is pivotal for monocyte survival and maturation, whereas macrophages with the low expression of *Cx3cr1* feature anti-inflammatory properties, showing the high plasticity of this macrophage subpopulation.^27^ An increased *Cx3cr1* mRNA expression in the sciatic nerve of EAN rats was detected at the later stages of EAN compared with the control group (26 dpi: *P*-value = 0.0286; 29 dpi: *P*-value = 0.0286). A similar expression pattern of marker *Mgl1* was detected (26 dpi: *P*-value = 0.1143; 29 dpi: *P*-value = 0.0286). *Mgl1* was previously tested in experimental autoimmune encephalomyelitis (EAE) mice and associated with anti-inflammatory effects and T-cell apoptosis.^28^ Therefore, an upregulation in the later stages of EAN was expected. Indeed, the highly increased *Mgl1* expression on Day 29 p.i. coincided with the strong upregulated *Il10* expression detected at the same time point. *Pf4*-expressing macrophages were evaluated for the first time in EAN and were upregulated at the latest examined time point (29 dpi: *P*-value = 0.0286) and showed no alterations at the maximum of EAN symptoms. The dynamic regulation of macrophage subpopulations indicates an interplay of pro-inflammatory and anti-inflammatory macrophages over EAN progression. It unmasks, in correlation to the persisting upregulation of pro-inflammatory and anti-inflammatory cytokines and cells on Day 29 p.i., a residual immunological adaptation at the molecular level.

### Involvement of small fibres

#### Development of hyperalgesia

**Figure 6:**
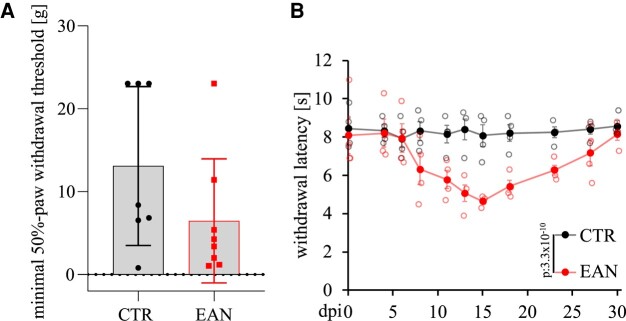
**Paw withdrawal measurement tests in EAN.** (**A**) Representative results of the vF hair test on Day 15 p.i. show a decreased minimum of paw withdrawal threshold. Each data point represents one animal. (**B**) A decreased withdrawal latency of EAN rats in the Hargreaves test with a monophasic progression was detected (*P*-value < 0.0001). Animals tested for the vF hair test include rats from the Peak and Recovery setups (EAN *n* = 8, CTR = 7). The Hargreaves test was performed with a *n* = 4/group. Each filled data point represents the mean of the group. The blank points are the respective animals of the group.

A previous study from our group determined the involvement of small nerve fibres in the EAN by detecting alterations in nociception, more precisely, mechanical hyperalgesia over the disease course.^21^ The performed Hargreaves and vF hair tests depicted an acute pain oversensitivity to mechanical and thermal stimuli, reaching its peak concordant with motor symptoms on Day 15 p.i. (Hargreaves test: *P*-value < 0.0001; Fig. 6). Hyperalgesia developed before the impairment of motor functions, with the first symptoms arising on Day 8 p.i. Complete recovery was achieved on Days 26 and 29 p.i. for the vF hair and Hargreaves tests, respectively.

#### TRPV1 as a possible determinant of hyperalgesia

**Figure 7:**
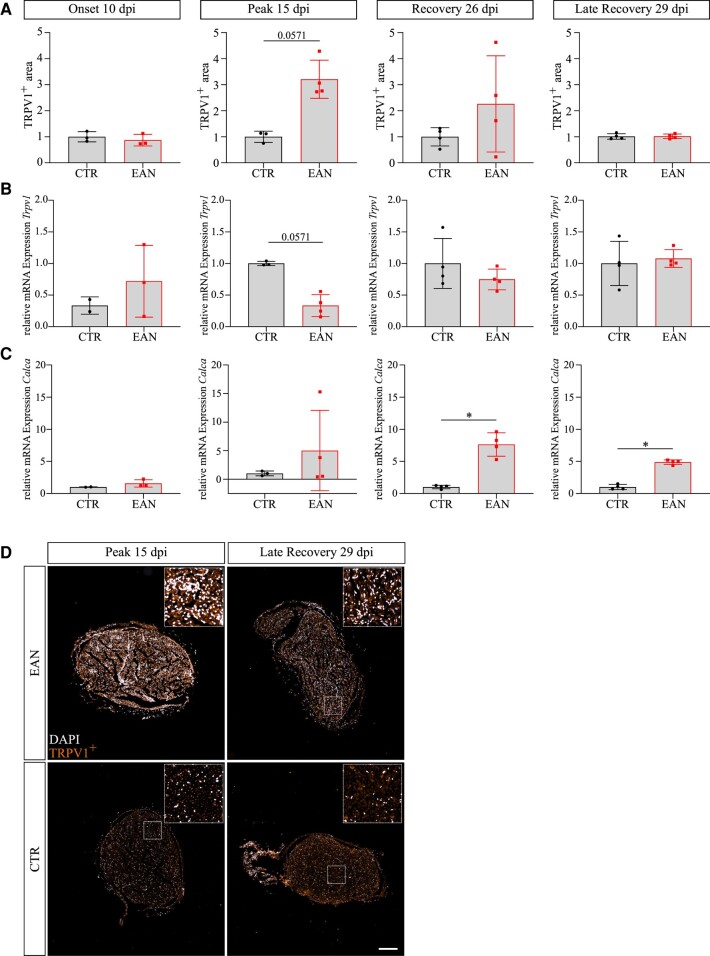
**TRPV1 and *Calca* expressions in the sciatic nerve of Lewis rats.** TRPV1 antibody was used to stain the vanilloid receptor 1 in nerve tissue and statistically analysed via Welch’s *t*-test or Mann–Whitney test. (**A**) TRPV1+ area is increased at the peak (*P*-value = 0.0571) of the EAN before recurring to the original state on Day 29 p.i.. Data points represent the TRPV1 area of the sciatic nerve in an animal. (**B**) The relative expression of *Trpv1* is downregulated at peak in the EAN (*P*-value = 0.0571). (**C**) Relative *Calca* expression shows an upregulation at recovery (*P*-value = 0.0286) and late recovery (*P*-value = 0.0286) in EAN rats. Quantitative PCR was performed with duplicates per animal and according to the normality distribution analysed with Welch’s *t*-test or Mann–Whitney test. Each data point represents an animal. (**D**) Representative pictures of TRPV1 staining in the sciatic nerve of Lewis rats. The scale indicates 200 µm.

Transient receptor potential (TRP) ion channels are known for the modulation of sensory sensation. TRPV1 is considered a primary receptor for nociception (mechanical, chemical and thermal).^29,30^ Immunohistochemical staining of sciatic nerves of EAN-affected rats showed an upregulation of TRPV1 in the endoneurium at peak coinciding with the severity of hyperalgesia (dpi 15, *P*-value = 0.0571). Afterward, the increased protein expression is reduced until there are no alterations detectable on Day 29 p.i. The mRNA expression of *Trpv1* in the sciatic nerves depicted a converse course with a compensatory downregulated expression on Day 15 p.i. (*P*-value = 0.0571) and subsequent gradual normalization. No changes were assessed in mRNA and protein expression on Days 10 and 29 p.i. The TRPV1 downstream molecule Calcitonin-gene related peptide (CGRP) was analysed at the mRNA level (*Calca*). The mRNA expression of *Calca* was upregulated at all examined time points, significant on Days 26 (*P*-value = 0.0286) and 29 p.i. (*P*-value = 0286), providing further indicators for the involvement of TRPV1 in the altered nociception (Fig. 7). TRPV1 staining in DRGs showed no alterations (data not shown), which implies an axonal or inflammatory origin of TRPV1.

### Integrity of small nerve fibres

#### Epidermal nerve fibres

**Figure 8:**
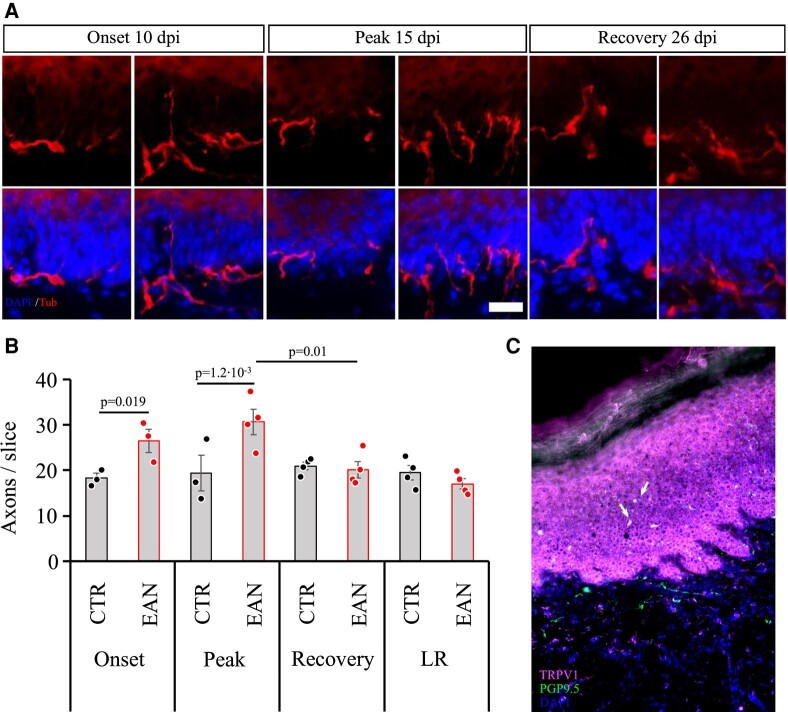
**Small fibres in the skin.** (**A**) Representative pictures of neuronal marker beta-III-tubulin staining plantar foot pads. (**B**) The amount of beta-III-tubulin–positive structures depicted in red in EAN rats is significantly higher at onset (one-way ANOVA followed by Tukey’s multiple comparison test: *P*-value = 0.02) and peak stage (*P*-value = 0.001). Each data point represents one animal. (**C**) Double-positive structures for the vanilloid receptor 1 TRPV1 (orange) and neuronal marker PGP9.5 (green) were observed. Nuclei were stained with DAPI and depicted in blue. The scale indicates 50 µm.

In the context of EAN, it is known that large nerve fibres are majorly affected. Still, there is a lack of studies regarding the integrity of the small fibres, which depicts a crucial part of the pathophysiology of human neuritis. Small fibre nerves in the plantar skin were stained and quantitatively analysed to investigate this matter. A significant increase of nerve fibres was detected in the stratum spinosum of EAN rats at the peak of the disease (*P*-value = 0.001), which also coincided with the high TRPV1 expression in the large fibres and the clinical hyperalgesia. The significantly elevated innervation was also present on Day 10 p.i. (*P*-value = 0.02). No alterations were detected at the later examined time points (Days 26 and 29 p.i.; Fig. 8B). Based on the previously mentioned hyperalgesia and dynamic TRPV1 regulation, the skin was stained against TRPV1 and PGP9.5 as an axonal marker. Double-positive axonal structures were observable (Fig. 8C). Furthermore, cells like keratinocytes were stained positive for TRPV1. The majority of PGP9.5^+^ structures, however, were TRPV1 negative; therefore, we conclude that TRPV1 changes are central in the large fibres and indirectly influence small fibres in the skin.

## Discussion

The purpose of this study was to provide an extensive characterization of EAN progression in Lewis rats, in particular, a correlation of inflammation through different immune populations with demyelination and extensive-fibre and small fibre pathologies.

We incorporate for the first time novel aspects, which have not been characterized before, such as small fibre involvement and pain sensation, bridging EAN pathophysiology to main symptoms of the human autoimmune neuritis as well as the role of specific macrophage populations.

Thus, the motor and sensory deficits of EAN animals were analysed for the first time via the CatWalk XT. This novel method allowed us to evaluate further potential clinical manifestations not registered by the classical scoring system and has proven to be a reliable method for scoring the EAN. We identified a prolonged remission of parameters until Day 29 regarding the paw integrity, implicating possible sensory ataxia. Sensory ataxia is caused by the impairment of the large myelinated somatosensory nerves, an aspect hardly investigated until now in EAN. Therefore, a thorough examination of disease recovery in EAN should include clinical evaluation for remaining sensory damage.

The involvement of the small nerve fibre and altered pain sensation was previously detected in the EAN by our group,^21^ and we reproduced these results in our current experiments. Changed integrity of small nerve fibres is associated with nociception. Concordant with previous findings of altered pain-like perception (nociception), our EAN rats experienced acute hyperalgesia.^20^ Interestingly, we found an increased innervation of the footpads already in the early and peak stages of the EAN. Studies on GBS and CIDP patients report however a reduced epidermal nerve density in skin biopsies with evidence of axonal degeneration.^31,32^ In these studies, skin biopsies were performed at a late disease stage with chronic axonal damage correlating with hypaesthesia. On the other hand, our group has conducted a prospective registry study and showed that almost 50% of CIDP patients present with neuropathic pain indicative of hyperesthesia.^33^ Therefore, small fibre involvement is evident in human and animal immune–mediated neuropathies, probably depending on the form and chronicity of the disease.

Our current EAN data imply for the first time that a ‘compensatory’ increase of skin innervation already at the EAN induction phase mediates this hyperesthesia. The mechanisms underlying this compensatory increase of skin innervation constitute critical implications for neuropathic pain research and treatment. One possible cause for the enhanced cutaneous innervation could be the elevated level of the nerve growth factor (NGF).^34^ Reduced availability of NGF is linked to the pathogenesis of the neuropathies,^35^ since NGF promotes axonal regeneration. Elevated NGF levels were detected in CIDP patients.^36^ This assumption and the findings of the skin biopsies correlate with our DRG outgrowth studies, which identified a higher potential for regeneration in EAN rats at the peak and recovery phases. Thus, the involvement of growth factors inducing compensatory nerve growth in this context should be investigated in more detail.

On the other hand, the skin in atopic dermatitis (AD) lesions is hyperinnervated with increased CGRP-positive nerve fibres.^37^ AD patients experience skin pain resembling neuropathic pain, which can be found in CIDP and GBS patients.^38^ CGRP is a molecule activated by TRPV1 signalling pathways. TRPV1 is a non-selective cation channel expressed in various cell types of the nervous system and immune system and is assumed to be a primary contributor to the pain sensation.^29,39^ We identified increased TRPV1 levels in the sciatic nerve via immunohistological staining at the peak phase of the EAN and a significant upregulation of the CGRP gene *Calca* at the recovery phase. Studies were able to link TRPV1 directly to inflammatory hyperalgesia, furthermore indicating that TRPV1 signalling pathways play a considerable part in EAN-induced hyperalgesia.^40^

Our group has previously shown the ameliorating effect of TRPV1 agonist capsaicin on EAN disease severity in a preventive concept^41^ and anti-oxidative effects in cell culture.^12^ Due to the complexity of TRPV1 signalling pathways, it is possible that TRPV1 influences both pain sensation and inflammatory modulation in EAN. Our group currently investigates capsaicin’s effect on sensory deficits and pain in the context of EAN, whereas capsaicin is already used through transdermal application for neuropathic pain.

EAN is known to alter the integrity of large nerve fibres primarily through demyelination.^14,42-46^ The clinical manifestations and, thus, the scores coincide with the severity of myelin degradation detected via immunohistochemistry and NCSs. Data for early axonal damage are scarce in EAN studies. NCSs could imply axonal damage through a distal CMAP reduction. However, no alteration of the GAP43 expression, a common marker for axonal degeneration/regeneration, in sciatic nerves at any time point was detected. In contrast, qPCR results showed a dynamic expression of *Gap43* over the whole course of the EAN, and this correlates with the regenerative potential of DRG in *ex vivo* studies (axonal growth). We assume that the changes in the regulation of *Gap43* are caused by Schwann cells. Schwann cell precursors and mature non-myelinating Schwann cells are known to express GAP43.^47^ Myelinating Schwann cells can be reformed into a non-myelinating type due to demyelination or axonal damage, providing a possible explanation for the dynamic expression of *Gap43* over EAN progression.^48^ Interestingly two rats showed an upregulation of SCG10, a regenerative marker of sensory axons at the recovery phase, indicating a previously occurring axonal damage. However, these changes were detected in only a minority of EAN rats, allowing no explicit statement regarding the progression and occurrence of axonal damage in the EAN. These findings support the general assumption of the EAN with a primarily demyelinating pathophysiology with secondary degeneration of axons and a regenerative potential through *Gap43,* confirmed through our DRG outgrowth experiment.

The main effector mechanism of EAN is the infiltration of T cells and macrophages into the peripheral nervous system (PNS). Our results circumstantiate this fact and proceed to a further characterization of macrophage pathology. The drastic changes in cell numbers from onset (10 dpi) to peak (15 dpi) illustrate the rapid process of infiltration into the sciatic nerves of EAN rats, which is also mirrored in the upregulated expression of the pro-inflammatory cytokine IFNg. The number of T cells decreases again after the peak of the disease, whereas the macrophages persist in higher numbers in the nerves. This is most likely due to the conversion of the pro-inflammatory M1 type of macrophages into the anti-inflammatory M2 phenotype. The M1 macrophages contribute to the inflammatory processes in the EAN by enhancing the permeability of the blood–nerve barrier, acting as antigen-presenting cells promoting Th1 polarization and autophagy of myelin.^49,50^ M2 macrophages exhibit neuroprotective functions in inflammatory diseases by promoting T-cell apoptosis, downregulation of inflammatory cytokines and clearance of debris.^51,52^ The detected remaining high number of infiltrated macrophages at the recovery phase combined with the amelioration of the clinical manifestations and electrophysiological properties and remyelination supports the hypothesis of the M1/M2 macrophage paradigm in the EAN.^53,54^ We proceed to answer the question of M1 and M2 macrophage markers in EAN. The identified macrophage subpopulations (PF4, MGL1 and CX3CR1) show a dynamic expression over the course of the EAN. MGL1-expressing macrophages were previously investigated in the model of EAE, where they exhibited anti-inflammatory effects and were linked to the secretion of interleukin-10.^28^ Our results show, indeed, a correlation between *Mgl1* and anti-inflammatory *Il10* expression. Especially the Day 29 p.i. is of interest since both *Mgl1* and *Il10* show a significant upregulation suggesting a polarization of a Th2 response even though all motor and sensory impairments are recovered. A possible explanation for this phenomenon is the potential onset of tolerance mechanisms/anti-inflammatory niche resulting in the monophasic EAN progression instead of a chronic course. Based on the literature, CX3CR1+ macrophages exert pro-inflammatory or anti-inflammatory effects depending on the expression level of CX3CR1. C*x3cr1* expression is increased over the whole progression of EAN, pointing to the pro-inflammatory and anti-inflammatory properties of the respective macrophages. PF4-expressing macrophages are associated with pro-inflammatory factors in later stages of diseases, such as Influenza A virus–driven pulmonary disease.^55^ However, not much in the context of neuronal autoimmune diseases is known. A significant upregulation in the relative mRNA expression of *Pf4* was found at the end of the recovery phase on Day 29 p.i. in EAN rats, hinting at a secondary wave of pro-inflammatory mediators. The assessed mRNA upregulation is not reflected in the upregulation of *Ifng* on mRNA level nor in a re-development of motor deficits. This suggests that either the pro-inflammatory aspects of PF4-expressing macrophages are suppressed, for instance, by the determined upregulation of anti-inflammatory mediators, such as MGL1 macrophages, or that the PF4 macrophage subset does not exhibit pro-inflammatory aspects in the EAN and could be a marker for M2 macrophages.

## Conclusion

EAN as a model for acute human immune neuropathies was traditionally regarded as a primary demyelinating disease with implicated secondary axonal damage, sensory impairment through recovery and a crucial role of macrophages for the effector and recovery phases. Our study showed novel EAN aspects, such as the affliction of small nerve fibres and their outgrowth of the plantar foot pad, a variety of macrophage M1 and M2 subpopulations and TRPV1 as a critical player for thermal and mechanical hyperalgesia. Further experiments must be conducted to further assess the underlying mechanism behind the altered integrity of the small nerve fibres and the respective inflammatory properties of the macrophage subsets. These novel aspects may have direct therapeutic implications for patients with neuropathic pain and the improvement of the immune system reconstitution through the recovery phase.

## Supplementary Material

fcae059_Supplementary_Data

## Data Availability

The data of this study are available from the corresponding author upon reasonable request.
