# Supplementary material for: Small fibre integrity and axonal pathology in the rat model of experimental autoimmune neuritis
Source: Brain Commun. 2024 Mar 1;6(2):fcae059. doi: 10.1093/braincomms/fcae059 (PMC10935649; doi:10.1093/braincomms/fcae059)
Supplement: fcae059_Supplementary_Data [file fcae059_supplementary_data.docx]

Supplementary Figure 1


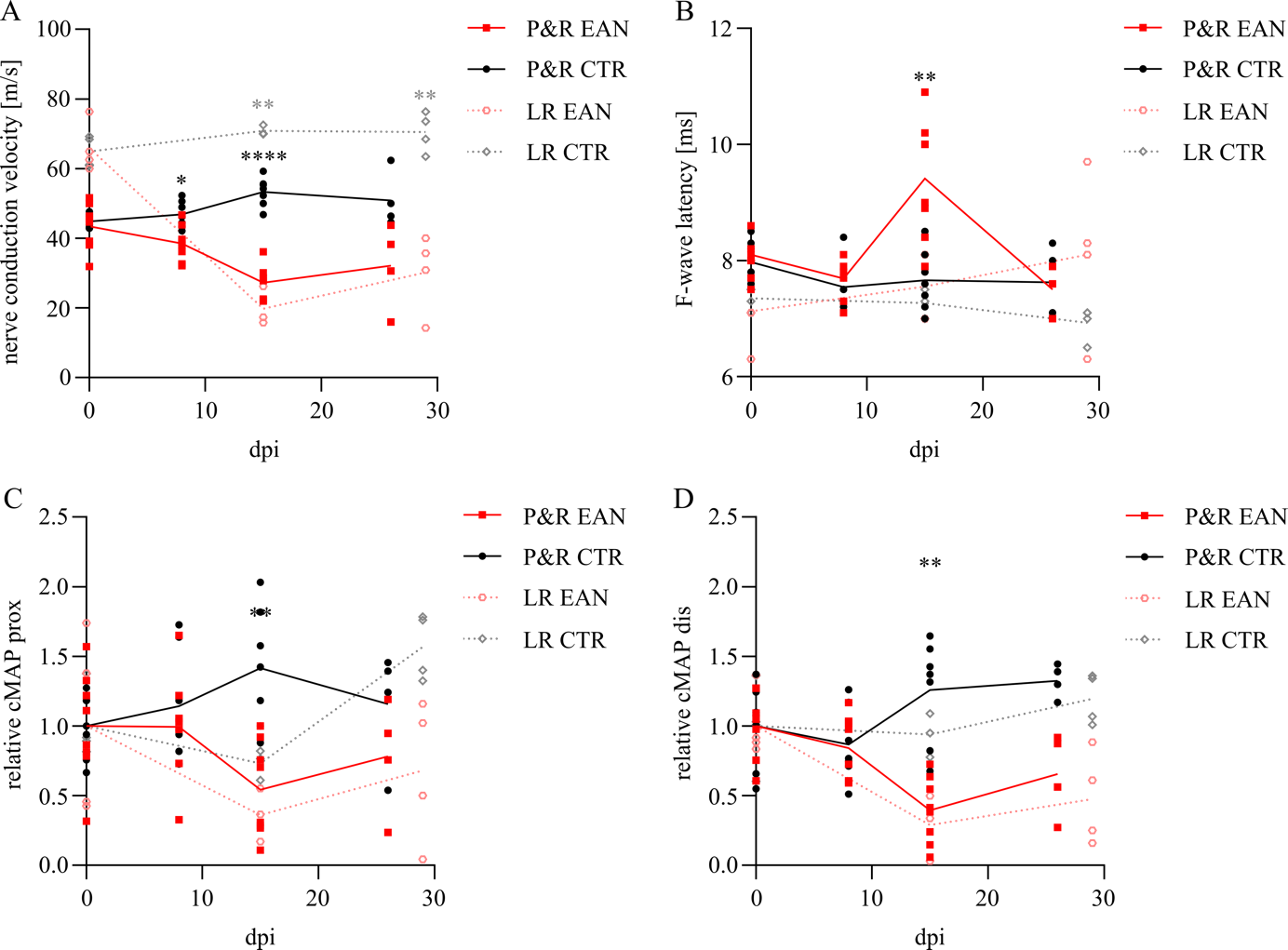


Supplementary figure 1:

MNCV, F-wave latency and CMAP analysis on sciatic nerves of EAN and CTR rats. Mixed-effect analysis followed by Tukey’s multiple comparison test were performed. EAN rats showed a significant deterioration of MNCV on day 8 (P-value = 0.0127) and 15 p.i. (P-value < 0.0001) (A) compared to the values at day 0. F-wave latency is significantly increased at peak time point in the EAN (P-value = 0.0056) (B). Distal and proximal CMAPs declined at 15 d.p.i. (P-value_proximal_ = 0.0013; P-value_distal_ = 0.0042) before partial recovery. Rats in the experiment “Late Recovery” were viewed separately due to the variance of the values measured in the other set ups (n = 4 / group). Day 0 p.i. includes a n = 11 / group, 8 p.i. = 11 / group, 15 p.i. n = 8 / group, 26 p.i. n = 4 / group. CTR = control; EAN = experimental autoimmune neuritis; dpi = day post immunization; P & R = Peak and Recovery; LR = Late Recovery; prox = proximal; dis = distal.

Supplementary Figure 2


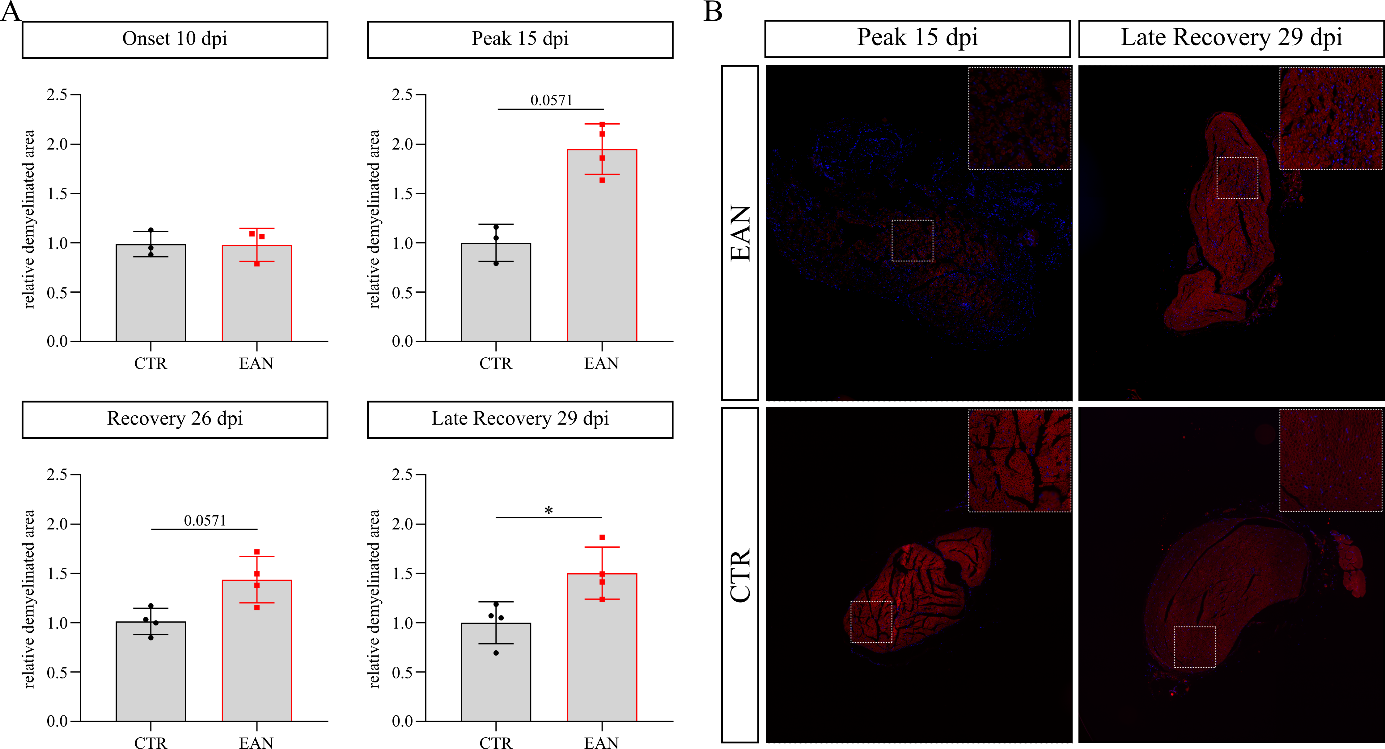


Supplementary figure 2:

Relative demyelinated area is significantly increased over the EAN disease progression (n = 4 / group) (A). Representative pictures of FluoroMyelin^TM^- Red staining in sciatic nerve of Lewis rats at peak and late recovery stage (B). Based on the normality either Welch’s t test or Mann-Whitney tests were performed. EAN rats at peak time point (P-value = 0.0571) exhibit a strong demyelination whereas the sciatic nerve of EAN rats at day 29 p.i. (P-value = 0.0286) shows remyelination to a certain extent. Scale indicates 200 µm. CTR = control; EAN = experimental autoimmune neuritis; dpi = day post immunization.

Supplementary Figure 3


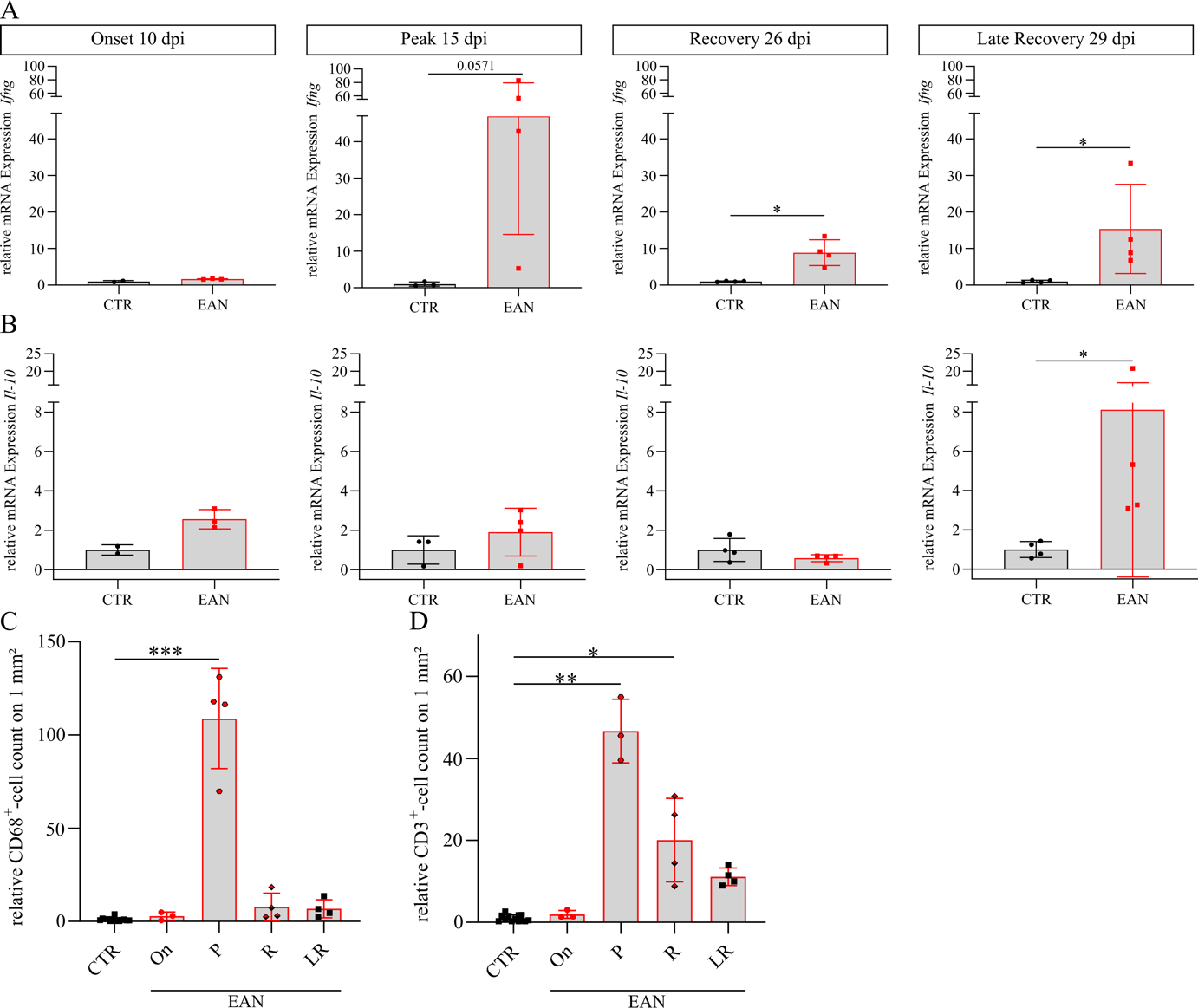


Supplementary figure 3:

Relative expression of *Ifng* and *Il10* and immune cell infiltration. Increase in the relative *Ifng* expression were detected at day 15 (Mann-Whitney test: P-value = 0.0571), 26 (P-value = 0.0286) and 29 p.i. (P-value = 0.0286) in EAN rats with the highest fold at peak time point (A). EAN animals exhibit a significant upregulation of *Il10* expression at day 29 p.i. (P-value = 0.0286) (B). Cell numbers of CD3^+^ cells (C) and CD68^+^ in the sciatic nerve show the EAN characteristic infiltration of T-cells and macrophages into the PNS (Kruskal-Wallis test followed by Dunnett’s multiple comparison; (CD3: P-value_peak_ = 0.0008; CD68: P-value_peak_ = 0.0034). CD68^+^ cells remain in higher numbers in the tissue of EAN animals (P-value_recovery_ = 0.0248). CTR = control; EAN = experimental autoimmune neuritis; dpi = day post immunization; P = Peak; R = Recovery; LR = Late Recovery.

Supplementary Figure 4


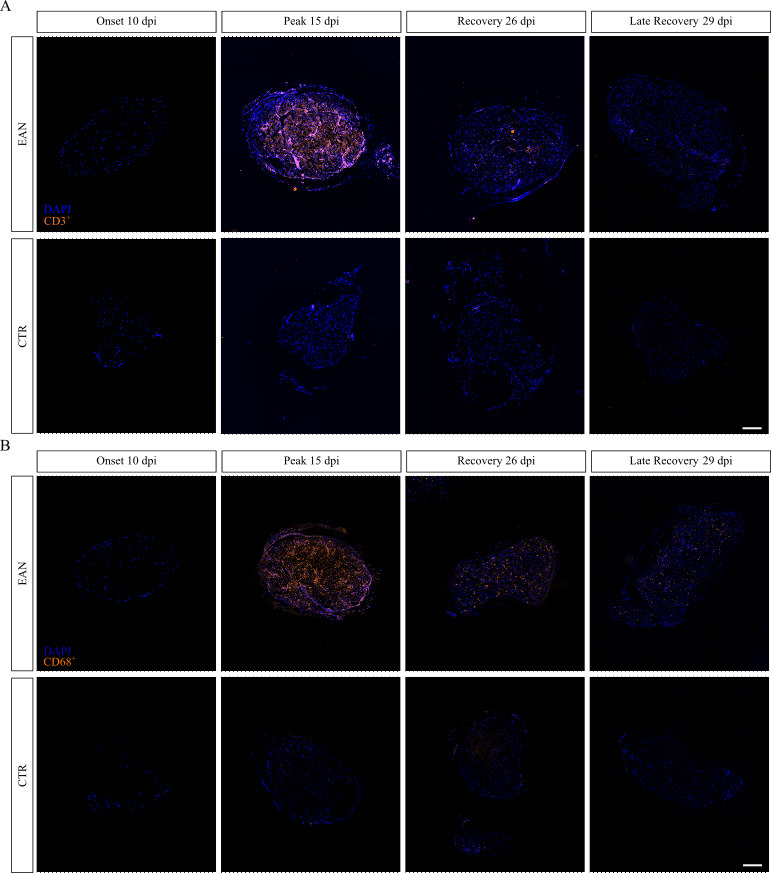


Supplementary Figure 4:

Representative picture of CD3^+^ cells (A) and CD68^+^ cells (B) in the sciatic nerve of Lewis rats show a high infiltration rate at peak in EAN rats with a subsequent decrease. CD68^+^ cells remain prolonged in the sciatic nerve. Scale indicates 200 µm. CTR = control; EAN = experimental autoimmune neuritis dpi = day post immunization.

**Supplementary Table 1**: targets and primer sequences for a quantitative real-time polymerase chain reaction

| Target | Sequence |
| --- | --- |
| Reference genes |  |
| *Actb*  (Beta-actin) | Sense: CCC ATC TAT GAG GGT TAC GC  Anti-sense: TTT AAT GTC ACG CAC GAT TTC |
| *Gapdh*  (Glyceraldehyde 3-phosphate dehydrogenase) | Sense: AGG TCA CCC AGA GCT GAA CG  Anti-sense: CAC CCT GTT GCT GTA GCC GTA T |
| Target genes |  |
| *Calca* (Calcitonin gene-related peptide) | Sense: CCT TCG GGT CTG AGG AAC TA  Anti-sense: GGC GTG GTG AGT TCA ACT TT |
| *Mgl1 (Clec10a)*  (Macrophage galactose type lectin 1) | Sense: ATG TGT ACT CCT GGC TTC TGC  Anti-sense: CTG AGG TAA GTC CTG GAA CTG G |
| *Cx3cr1*  (CX3C chemokine receptor) | Sense: CTG CAG AAG TCC CCG TCC  Anti-sense: AAA GGC CAC GAT GTC ACC C |
| *Gap43*  (Growth associated protein 43) | Sense: CTC TCC TGC CCT TTC TCA GAT  Anti-sense: ACT CGC CAT AAC AAC ACC AAG |
| *Ifng*  (Interferon γ) | Sense: AAA GAC AAC CAG GCC ATC AG  Anti-sense: CTT TTC CGC TTC CTT AGG CT |
| *Il10*  (Interleukin-10) | Sense: CCT GCT CTT ACT GGC TGG AG  Anti-sense: TCT CCC AGG GAA TTC AAA TG |
| *Pf4*  (Platelet factor 4) | Sense: GTT CCC CAG CTCATA GCC AC  Anti-sense: TTA TAC AGA GGT ACT TGC CGG TC |
| *Trpv1*  (Transient receptor potential vanilloid 1) | Sense: CTT CTG AGG GAT GCA AGC AC  Anti-sense: CCT GGG ACC ATG GAA TCC TT |
